# Gas Barrier, Thermal, Mechanical and Rheological Properties of Highly Aligned Graphene-LDPE Nanocomposites

**DOI:** 10.3390/polym9070294

**Published:** 2017-07-21

**Authors:** Karolina Gaska, Roland Kádár, Andrzej Rybak, Artur Siwek, Stanislaw Gubanski

**Affiliations:** 1 Department of Electrical Engineering, Chalmers University of Technology, 41296 Gothenburg, Sweden; stanislaw.gubanski@chalmers.se; 2 Department of Industrial and Materials Science, Chalmers University of Technology, 41296 Gothenburg, Sweden; roland.kadar@chalmers.se; 3 ABB Corporate Research Center, 31038 Krakow, Poland; andrzej.rybak@pl.abb.com (A.R.); artur.siwek@pl.abb.com (A.S.)

**Keywords:** polymer-matrix composites, rheological properties, thermal properties, permeability, extrusion

## Abstract

This contribution reports on properties of low-density polyethylene-based composites filled with different amounts of graphene nanoplatelets. The studied samples were prepared in the form of films by means of the precoating technique and single screw melt-extrusion, which yields a highly ordered arrangement of graphene flakes and results in a strong anisotropy of composites morphology. The performed tests of gas permeability reveal a drastic decrease of this property with increasing filler content. A clear correlation is found between permeability and free volume fraction in the material, the latter evaluated by means of positron annihilation spectroscopy. A strong anisotropy of the thermal conductivity is also achieved and the thermal conductivity along the extrusion direction for samples filled with 7.5 wt % of GnP (graphene nanoplatelets) reached 2.2 W/m·K. At the same time, when measured through a plane, a slight decrease of thermal conductivity is found. The use of GnP filler leads also to improvements of mechanical properties. The increase of Young’s modulus and tensile strength are reached as the composites become more brittle.

## 1. Introduction

Gas-barrier properties of polymers have become essential in various fields, like in packaging, pharmaceutical applications and in electronics. Polymeric materials gradually replace conventionally used metals and paper in the packaging industry thanks to the ease of their processing, light weight, low costs as well as multifunctional characteristics [1]. However, these applications are often limited because of relatively high permeability for gases. A number of studies on the enhancement of gas-barrier properties in polymeric materials have thus been carried out, while striving to maintain good mechanical properties and processing characteristics for meeting the needs of modern industrial applications. Among these is also a possible use of polymeric gas barriers in high-voltage switchgear apparatuses, operating normally for long periods of time under pressurized conditions.

Sulfur hexafluoride (SF_6_) is used as an insulation gas in electrical equipment (e.g., gas-insulated switchgear) due to its superb dielectric properties. Recently new eco-efficient gas mixture media were developed, namely fluoroketone-based gas mixture with CO_2_ as a carrier gas [2]. The main shortcoming of SF_6_ is its high global warming potential, much higher, about 23,500 times, than for CO_2_. Gas leakage of SF_6_ is thus considered as high environmental pollution, namely having a strong effect on ozone depletion and as consequence global warming [3]. Therefore, its emission should be reduced.

As is commonly known, properties of polymers can be dramatically changed by incorporation of nanofillers. Manufacturing of polymer nanocomposites is nowadays crucial in order to secure development of high-performing and multifunctional generations of reliable materials. Graphene has also attracted much attention as a nanofiller candidate for polymer-based nanocomposites due to its unique thermal, electrical and mechanical properties [4,5,6,7,8,9]. Moreover, graphene and its modifications are considered among 2-D materials with high surface area of flakes and high aspect ratio as a potential candidate for creating efficient barriers to the permeation of gasses [10,11]. However, as the manufacturing of high amounts of defect-free, monolayer graphene is still challenging, graphene-nanoplatelets are a good source of this nanofiller for the production of bulky nanocomposites. Graphene nanoplatelets usually consist of several stacked graphene layers, which can be exfoliated into monolayer or a few-layer graphene nanofiller, which is expected to increase the tortuosity of gas diffusion through nanocomposite and, as a result, to extend a travelling pathway of the diffusing gas through the material layer. A similar effect have also been observed when incorporating nanoclays into polymer matrices [12,13]. Moreover, using graphene nanoplatelets as a filler results in modification of polymer chain mobility and, as a consequence, decreases available free volume within polymer matrix for diffusing gas molecules [14].

The recent investigations showed that incorporation of graphene nanoplatelets can reduce dramatically gas permeability [15,16,17,18]. The work by Cui et al. [11] showed recent development of the graphene application as a filler incorporated into different polymer matrices for barrier application. A proper dispersion and uniform distribution are the crucial issues to obtain the desired property. Therefore, achieving a high level of exfoliation and controlled nanoplatelet orientation are here the most important challenges. As the macroscopic properties the graphene nanocomposites strongly depend on the interfacial compatibility (polarity match) of polymer and filler particles [19], achieving this aim in polyolefin-based matrices is a challenging task that requires selection of a proper manufacturing technique [7]. Numerous attempts have been made [20,21] by using solution mixing, melt mixing and in situ polymerization [11]. Moreover, achieving high anisotropy in composite morphology is also a crucial issue. Various methods have therefore been applied with successful attempts by using magnetic or electric field-assisted methods to align the filler [22,23,24]. Many researchers have shown that composite morphology is strongly dependent on the polymer nature [25,26]. In the case of thermoplastic polymers, filler alignment can be obtained in situ during manufacturing process of nanocomposite, where a good example is the melt extrusion technique [27,28,29,30] or three-dimensional printing [31].

We thus report herewith on the use of melt extrusion process preceded by pre-coating compounding method for obtaining anisotropic nanocomposites, filled with exfoliated and well dispersed graphene nanoplatelets (GnP), for reduction of gas permeation ability while preserving or strengthening other technically important parameters of the composites. As to our knowledge, the presented here measurements of permeability of SF_6_ gas are original and unique in literature.

## 2. Materials and Methods

### 2.1. Materials

Low-density polyethylene (LDPE) was delivered by Borealis AB (Stenungsund, Sweden) in form of pellets and graphene nanopowder xGnP M5 were purchased from XG Sciences (Lansing, MI, USA). Properties of the graphene nanoplatelets and molecular weight parameters of the LDPE, measured by means of Gel Permeation Chromatography and DSC, are presented in Table 1.

### 2.2. Materials Processing

#### 2.2.1. Precoating Technique

**Figure 1:**
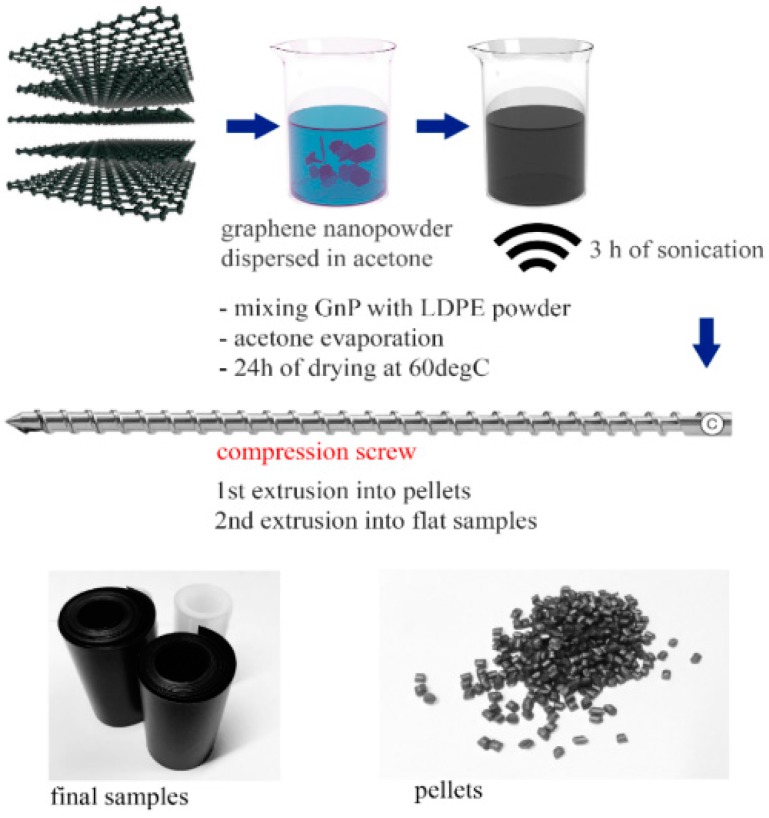
Scheme illustrating samples preparation process.

The manufacturing process of the studied specimens is illustrated in Figure 1. In the first step LDPE pellets were cryogenically grounded into a powder using high-speed rotor mill. The obtained this way average diameter of the powder particles was 0.5 mm. Thereafter, a modified coating technique, originally developed by Drzal group [32], was applied. Graphene nanoplatelets were dispersed in acetone and then placed in sonication bath for 3 h, 90 W. Low power sonication is here essential to eliminate agglomerates and to improve exfoliation of the nanopowder, without damaging its flakes [33,34,35]. Thereafter, LDPE powder was mixed with exfoliated graphene nanoplatelets in acetone, using an overhead stirrer rotating at 500 rpm for 40 min, until full evaporation of acetone. Obtained this way masterbatches were dried in an oven at 60 °C for 24 h.

#### 2.2.2. Melt Extrusion and Film Casting

Brabender 19/25 D (Brabender GmbH & Co, Duisburg, Germany) single-screw extruder (screw diameter *D* = 19 mm and a screw length of 25 D) equipped with conveyor belt was used. LDPE-GnP masterbatches were extruded twice by means of compression screw (CS, compression ratio 2:1), which secures distributive mixing. This mechanism is based on continuous rearrangement of composite constituents and yields high homogeneity of the manufactured material. The first extrusion process was treated here as melt compounding of the LDPE-GnP masterbatches, after which the obtained material was pelletized. Thereafter, a second extrusion process was carried out in order to obtain composite films with an average thickness of 0.1–0.3 mm. The extruder temperatures, from the hopper to the die, were respectively: 115, 130, 130 and 140 °C and a constant speed of 5 rpm was kept during the process. The produced samples varied in filler content as follows: 1, 3, 5 and 7.5 wt %.

### 2.3. Experimental Techniques

#### 2.3.1. Scanning Electron Microscopy

A FEI/Philips Field Emission Scanning Electron Microscope (Hillsboro, OR, USA) was used to investigate the morphology of the LDPE-GnP nanocomposites. The samples were cooled down in liquid nitrogen, and then fractured. Thereafter all samples were etched for one hour using solution of 1 wt % potassium permanganate in a mixture of sulfuric acid, ortho-phosphoric acid and water [36]. The process was terminated by cleaning in a mixture of sulfuric acid and water, thereafter in hydrogen peroxide and finally in isopropanol. This process has been applied only on samples used in SEM observations. The etching has been performed in order to show clearly the orientation of graphene nanoplatelets as well as filler concentration. The cryo-fracturing method is widely used to expose filler in thermoplastic nanocomposites. However, the alignment was not clearly visible when only cryo-fracturing has been used. Therefore the chemical etching has been applied in order to enhance visibility of oriented graphene nanoplatelets. Afterwards, approximately 5 nm thick gold layer was deposited onto the observed surfaces using a Sputter Coater S150B, BOC Edwards, Crawley, UK.

#### 2.3.2. Thermal Conductivity

Thermal conductivity was measured by means of Hot Disc Thermal Constants Analyser 2500 S (Hot Disk AB, Göteborg, Sweden). The principle of this method is described in [37] and the measurements were carried out according to ISO Standard 22007-2. The selection of software modules in the instrument allows the determination of thermal conductivity of anisotropic samples. The axial (through plane) and radial (in-plane) directions can be distinguished. All the measurements were carried out at room temperature.

#### 2.3.3. Rheological Properties

**Figure 2:**
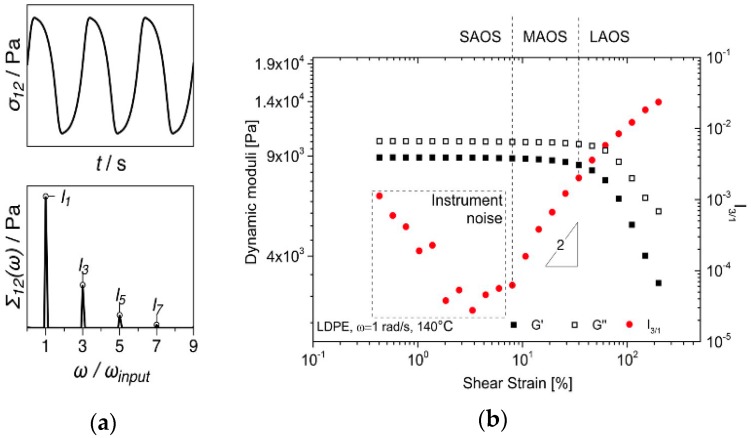
(**a**) Illustration of a nonlinear material shear stress response and the occurrence of (odd) higher harmonics in the corresponding Fourier spectra; (**b**) Variation of the dynamic moduli and third relative higher harmonic, *I*_3/1_, during a strain sweep test on the pure LDPE sample.

The rheological characterization of the nanocomposites was performed on an Anton Paar MCR702 TwinDrive rheometer (Anton Paar GmbH, Graz, Austria). A plate-plate geometry with a diameter of 25 mm and a gap of 1 mm was used. Linear and nonlinear viscoelastic oscillatory shear tests were performed with the purpose of determining the rheological percolation thresholds. In linear viscoelastic frequency sweeps, the rheological percolation can typically be detected via the storage modulus, *G*’, plateau recorded at low angular frequencies, signifying the additional elastic contribution of the filler network [38,39,40,41]. A more sensitive method to investigate rheological percolation is nonlinear viscoelasticity. Fourier-transform (FT) rheology was used for nonlinear viscoelastic measurements in large amplitude oscillatory shear (LAOS) sweeps. In a nonlinear viscoelastic material response, the imposed sinusoidal strain input signal results in a nonlinear periodic stress output signal. This, in turn, generates higher harmonics in the associated Fourier spectrum, see Figure 2a. One of the foremost advantages of this approach is the superior signal-to-noise ratio compared to linear viscoelastic measurements [42]. The influence of filler content and percolation was detected using the relative third higher harmonic of the shear stress response, *I*_3/1_ [43,44,45] as it contains the dominant nonlinear contribution to the signal [42]. A typical strain amplitude dependence of the third relative higher harmonic, *I*_3/1_, is illustrated in Figure 2b. At low strain amplitudes (SAOS—Small amplitude oscillatory shear), the dynamic moduli are in the linear viscoelastic regime and therefore independent of the applied strain amplitude. In this region the *I*_3/1_ response is dominated by instrumentation noise [40], whereby typically *I*_3/1_·α·γ*^−^*^1^. Generically, the limit of the linear viscoelastic regime (SAOS) is where the dynamic moduli are no longer independent of the applied strain amplitude. A more accurate change in material response is evidenced by the *I*_3/1_ nonlinear parameter. At the onset of the nonlinear regime, *I*_3/1_·α·γ^2^, region that is referred to as medium amplitude oscillatory shear (MAOS) [33] or intrinsic LAOS [34]. Thereafter, the LAOS (large amplitude oscillatory shear) regime is achieved, with *I*_3/1_ expected to level off at the high-strain amplitudes [43].

#### 2.3.4. Gas Permeation

**Figure 3:**
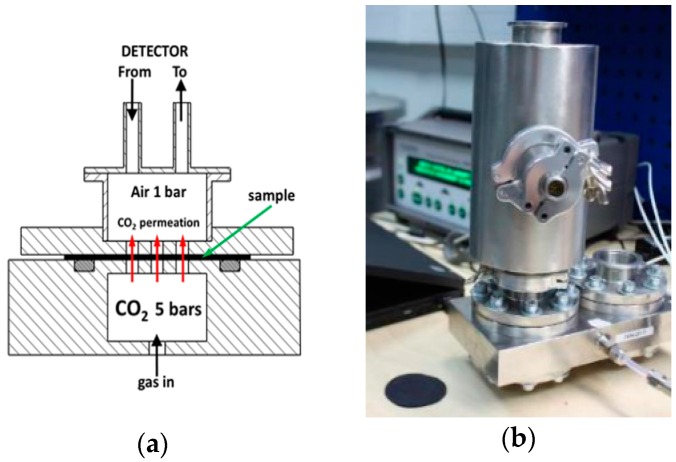
Schematic view (**a**) and photo (**b**) of the permeation setup (black circular sample is shown in left bottom corner).

CO_2_ and SF_6_ permeation rates were measured by monitoring the gas concentration in the closed volume of air under the atmospheric pressure. The gas was supplied from the other side of specimen under the pressure of 5 bars, as illustrated in Figure 3a. The measured sample was in the form of 0.3 mm thick plate mounted between two flanges. To avoid sample deformation or breakage, the surfaces of the flanges were used as a support, and 14 holes of 2 mm diameter allowed the gas to permeate to the upper volume under the atmospheric pressure, where its concentration was determined. Due to the high density of SF_6_, a small fan was placed in the measuring chamber to ensure its homogenous distribution and an accurate measurement of the leakage through the sample. For concentration measurements the LumaSense Photoacoustic Gas Monitor INNOVA 1412i (LumaSense Technologies, Inc., Santa Clara, CA, USA) was used. The sensitivity of this instrument is as low as 6 ppb. SF_6_ gas was fed with the small service unit of the “Micro” series from Dilo, allowing for both filling and complete recapture of the gas.

#### 2.3.5. Positron Annihilation Spectroscopy

Positron Annihilation Lifetime Spectroscopy (PALS) method was used in order to evaluate free volume fractions in the nanocomposites [46]. The positron lifetime (LT) spectra of the samples were performed using a fast–fast type spectrometer equipped with BaF_2_ scintillators which were connected to the photomultipliers XP2020Q (Photonis, Brive, France). Additional electronic units used in the spectrometer were: Constant Fraction Discriminator 583 (Ortec, Oak Ridge, TN, USA), Time Amplitude Converter 566 (Ortec, Oak Ridge, TN, USA), and Multichannel Analyzer ADC 8701 (CANBERRA, Meriden, CT, USA). The sodium isotope ^22^Na enveloped in 7 µm Kapton foil was used as positron source. Such prepared positron source was positioned between the surfaces of the two identical samples of the investigated nanocomposites. Then the sandwich-like testing sample was positioned in front of the scintillation detectors of the PALS spectrometer. The positron lifetime spectrum was measured for 24 h in order to obtain more than 10^6^ counts in the spectrum and as a result good signal to noise ratio. The time resolution (FWHM) of the lifetime spectrometer was 250 ps. Deconvolution of each spectrum was performed and the volume fraction of the free volume was obtained [47,48].

#### 2.3.6. Mechanical Properties

Mechanical properties of the composites were measured by means of Instron 5567 (Instron, Norwood, MA, USA) universal testing machine. Tensile tests were carried out according standard ISO 37-2, which describes a method for the determination of the tensile stress-strain properties of thermoplastic materials. Samples were cut along the extrusion direction and perpendicular by means of dumbbell cutter (Elastocon EP 04, Brämhult, Sweden) with overall length 75 mm and gauge length 20 mm. All the tests were performed at room temperature and the presented results are the average values of five replicable measurements.

## 3. Results and Discussion

### 3.1. Morphology of Nanocomposites

**Figure 4:**
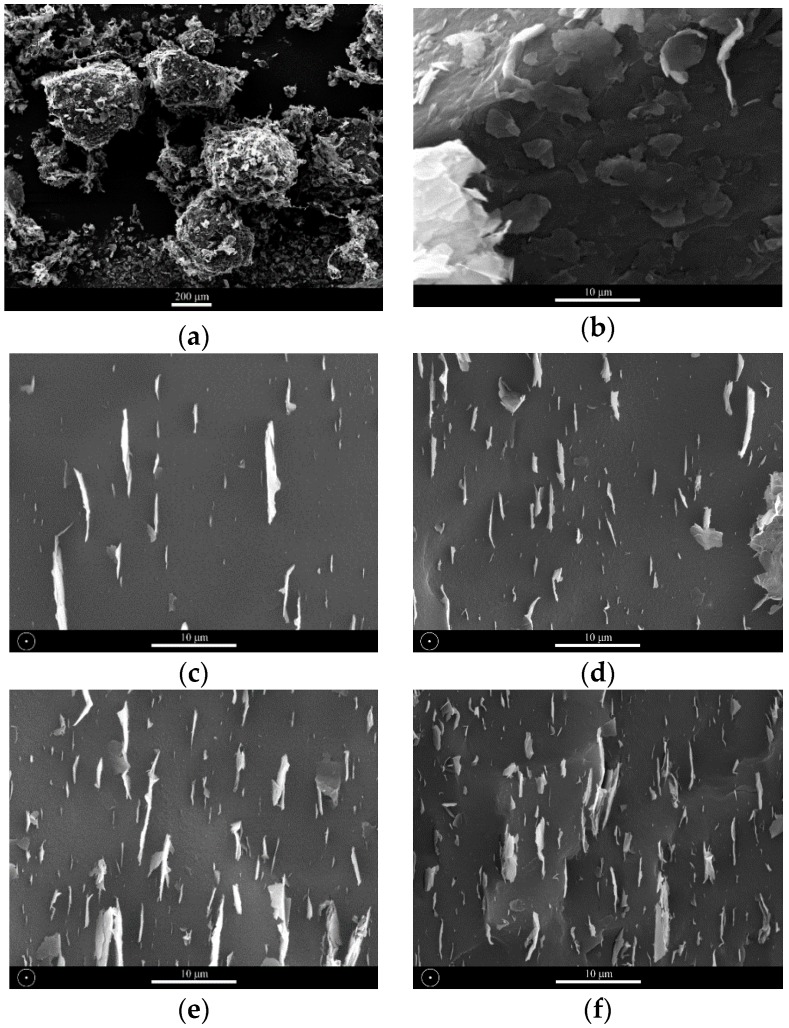
SEM images of LDPE powder coated with GnP (**a**,**b**) and freeze-fractured surfaces of nanocomposites filled with respectively 1%, 3%, 5% and 7.5% of GnP (**c**–**f**). Extrusion direction is indicated by circles.

The freeze-fractured surfaces of LDPE-GnP nanocomposites have been investigated by means of SEM to assess the dispersion of the filler. Photos in Figure 4 illustrate coverage of LDPE particles by GnP after precoating as well as morphology of freeze-fractured nanocomposite surfaces. The surfaces (photos c–f) are perpendicular to the processing direction and confirm uniformity of distribution of graphene flakes without visible agglomerated structures. The graphene nanoplatelets are oriented along polymer flow in the extrusion direction. It is known as flow-induced orientation, where apparent morphology arises generally during composite processing. The shear forces as well as extensional flow during extrusion process yield to the strong GnP alignment and as a result strong anisotropy in morphology.

Within the limits of the extrusion setup used in the present study, a preferential orientation of the nanoplatelets in the extrusion flow direction was observed starting with apparent die shear rates as low as 35 1/s (*n* = 5 rpm) [49]. Furthermore, SEM micrographs corresponding of “freeze-screw” experimental trials have shown that orientation along the flow streamlines could be observed as even in the metering screw region (compression screw, 2:1) with the nanoplatels oriented in the recirculation regions [27,49].

### 3.2. Thermal Conductivity

**Figure 5:**
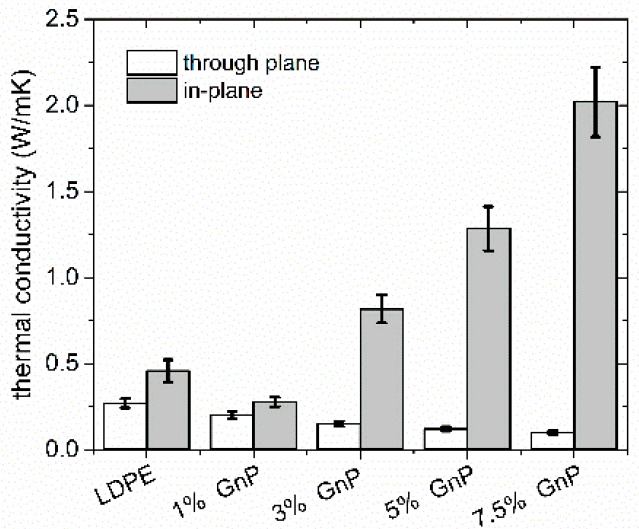
Thermal conductivity of nanocomposites measured in plane and trough plane direction.

Figure 5 shows thermal conductivity of the LDPE-GnP nanocomposites and of pure LDPE, as a reference. One can observe an increase of thermal conductivity along extrusion direction for all the measured and for the sample filled with 7.5 wt % of GnPs, the increase was as large as 388%. However, for samples measured through plane, a slight decrease of thermal conductivity was obtained. As expected, the strong anisotropy has been the reason, where creation of conductive paths along extrusion direction is very efficient. In general, thermal conductivity of the GnP nanocomposites depends on various factors, such as polymer morphology and its crystallinity, thermal conductivity of the polymer, thermal conductivity of GnP, GnP shape, flatness ratio, surface quality and levels of exfoliation and agglomeration [50,51]. In addition, one of the most important parameters is the quality of the interface between the polymer and the filler. As thermal energy in polymers and carbon fillers is conducted through phonon scattering processes, poor contact at the interface and weak bonding between GnP and polymer matrix leads to the increase of thermal resistance at the interface (Kapitza resistance *R*_K_) [52]. The resistance at the interface is related to the mismatch between phonon vibrational spectra of LDPE and GnP [52,53]. Therefore, interfacial scattering processes of phonons between GnP and LDPE are much stronger in perpendicular direction due to much bigger interface area as for parallel direction. Backscattering process of the phonons may also occur, yielding the decrease of effective thermal conductivity. Moreover, thermal conductivity of graphene flakes and graphite is well known as highly anisotropic (see Table 1). The strong *sp*^2^ bonding between carbon atoms causes high in-plane thermal conductivity, whereas through plane heat flow can be limited by weak van der Waals bonding. The anisotropy of thermal conductivity is however also visible also for reference pure LDPE sample and results from polymer chains stretch during the extrusion process [54].

### 3.3. Rheological Properties

**Figure 6:**
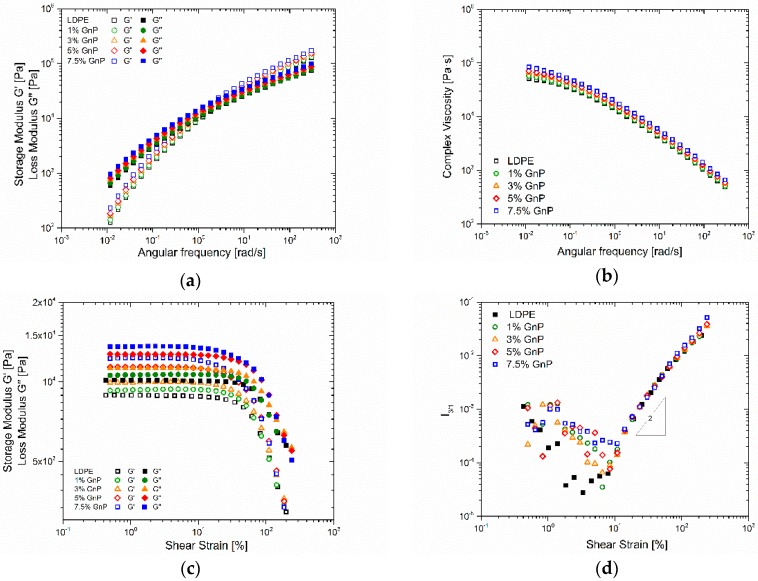
Frequency sweep results showing the dynamic moduli (**a**) and the complex viscosity (**b**); strain sweep results showing the dynamic moduli (**c**), and the relative third harmonic of the shear stress response, *I*_3/1_ (**d**).

Viscoelasticity measurements of LDPE-GnP nanocomposites were performed at 140 °C, which were carried out to elucidate the impact of GnP loading as well as to study the morphology and the formation of internal network within the manufactured composites. Storage modulus *G*’, loss modulus *G*” and complex viscosity dependence on the angular frequency in linear the linear viscoelastic regime are presented on Figure 6a,b respectively. When considering the moduli dependence in the terminal region, Figure 6a, a slight increase with the increasing GnP content in the whole angular frequency range is recorded. However, the existence of an additional elastic contribution in the lower limit of the angular frequency range cannot be clearly evidenced. Correspondingly, moderate increases in the complex viscosity are recorded, Figure 6b. Figure 6c shows the dynamic moduli dependence on the applied strain amplitude at constant angular frequency across the linear and nonlinear regimes. The corresponding relative third higher harmonic (*I*_3/1_) of the shear stress material response signal is shown in Figure 6d. For percolated filled polymer systems, a change in magnitude and in the scaling behavior of *I*_3/1_ during LAOS has been observed in similar studies [43,44]. Furthermore, it was shown that a change in the SAOS–MAOS transition region in the form of a plateau could be recorded for percolated systems, when compared to the reference polymer data [43]. It is important to point out that in nonlinear conditions the presence of a percolated network was shown using the nonlinear approach at angular frequencies that do not disclose such behavior in linear viscoelastic measurements. Results in Figure 6d show a weak change in scaling law in the LAOS regimes with increasing filer concentration. However, at potential plateau at the SAOS–MAOS transition region could be identified at 7.5% GnP as possible evidence for rheological percolation.

### 3.4. Gas-barrier Properties

**Figure 7:**
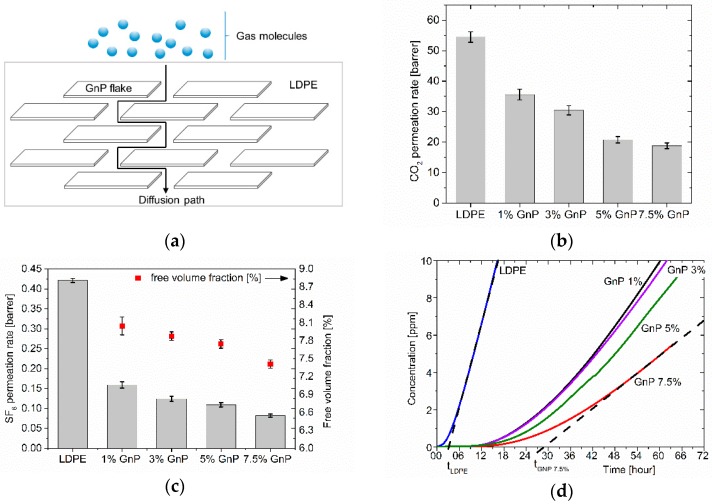
Schematically shown tortuous path of gas diffusion through a LDPE-GnP extruded nanocomposite (**a**); permeability of nanocomposites for CO_2_ (**b**); permeability of nanocomposites for SF_6_ and their free volume fractions (**c**); dependence of accumulated SF_6_ leakage (**d**) for LDPE-GnP nanocomposites concentration (*t*_LDPE_ and *t*_GNP7.5%_ indicate respectively delays in onset of permeation for pure LDPE and 7.5 wt % GnP samples).

Figure 7a illustrates schematically the tortuous diffusion path that can be created by means of GnP flakes in the investigated nanocomposites. It is expected that diffusion time of gas molecules through such membranes increases dramatically due to the fact that gas molecules are forced to travel around impenetrable filler flakes through more tortuous and complicated paths [11,13,55]. Moreover, gas-barrier properties depend on filler aspect ratio as well as the quality of its dispersion, level of agglomeration and orientation inside LDPE matrix. Structure of polymer matrix, namely its crystallinity level, has also an effect on gas-barrier properties as crystalline lamellas can hinder gas diffusion through polymer matrices [56].

Figure 7b presents the effect of GnP on permeability of CO_2_ through LDPE-GnP composites versus filler content and Figure 7c permeability of SF_6_ gas. As expected, the permeability of CO_2_ decreases gradually with increasing filler content. CO_2_ permeation decreased by 34.7% for sample filled with 1 wt % of GnP. Moreover, 65.5% reduction was achieved for sample with the highest GnP concentration. The obtained for CO_2_ level of permeability and reduction factor are in the same range as reported by for similar system in [18]. In the case of SF_6_ the permeability decreased dramatically for sample filled with 1 wt % of GnP content by 62.2% and by 80.5% for sample filled with 7.5 wt %. This behavior is related to the size of gas molecules, as molecules of SF_6_ are much larger than CO_2_ and the barrier effect is visible already at lower concentrations of GnP. It is also worth point out that the measurements of permeability of SF_6_ gas are original and unique in literature.

Additionally, a clear correlation between decrease of the permeability and decrease of the free volume fraction related to incorporation of GnP can be seen in Figure 7c. As already mentioned, GnP addition results in modification of polymer chains mobility and the free volume fraction within polymer matrix is reduced, which makes the material less permeable for gases.

Figure 7d presents the accumulated leakage of SF_6_ versus time. The influence of the filler content is visible and the sample with 7.5 wt % GnP delayed significantly the leakage in comparison to the reference LDPE sample by almost 25 h.

### 3.5. Mechanical Properties

**Figure 8:**
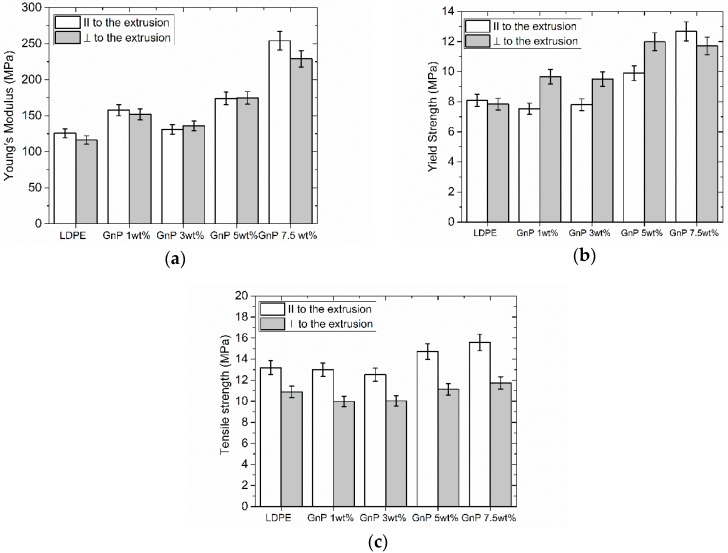
Comparison of Young’s modulus (**a**), Yield strength (**b**) and (**c**) tensile strength for LDPE-GnP nanocomposites.

Figure 8 presents results of Young’s modulus (a), yield strength (b) and tensile strength (c) measurements for samples cut perpendicular and parallel to the extrusion direction.

One can observe that Young’s modulus increases in both directions, with 102% increase for the sample filled with 7.5 wt % in parallel direction and 93% increase in perpendicular direction. Yield strength also increases with increasing filler content. One may also see that tensile strength remains at the same level for samples cut perpendicular to the extrusion direction and slightly increased for samples cut parallel to the extrusion direction. It can thus be concluded that there is no significant change in mechanical properties. This behavior could be explained by existence of wrinkled and not fully exfoliated GnP, influencing the stiffness of the composites.

**Figure 9:**
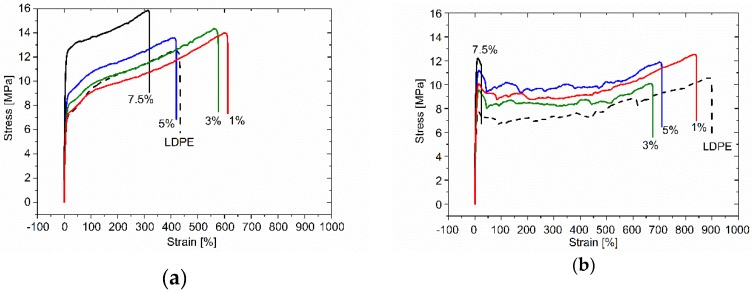
Stress-strain curves measured for reference LDPE and LDPE-GnP nanocomposites; parallel to the extrusion direction (**a**) and perpendicular to the extrusion direction (**b**).

Figure 9 illustrates comparison of stress–strain curves for neat LDPE as compared with the nanocomposites samples, showing the impact of extrusion [27,57]. As the shear stress applied during the processing leads to strong alignment of GnP flakes along extrusion direction as well as orientation of polymeric chains, the maximum strain in both directions (in extrusion direction and perpendicular to it) decreases while the tensile strength increases with increasing filler content (see also Figure 8). This behavior is related to the decrease of available free volume as well as polymer chains mobility. In consequence the composites become more rigid.

## 4. Conclusions

Nanocomposites of LDPE filled with GnP produced by melt extrusion process possess good filler dispersion as well as strong anisotropy in its orientation. This specific morphology leads to the creation of tortuous paths for permeation gas molecules through the nanocomposite bulk. A strong anisotropy in thermal conductivity is also achieved, showing a strong increase of thermal conductivity along extrusion direction. At the same time, thermal conductivity measured through-plane decreases slightly, mainly due to strong phonon scattering at the nanofiller interfaces. GnP nanofiller also leads to the improvement of mechanical properties, as manifested by the increase of Young’s modulus and tensile strength. Stress-strain curves demonstrate increasing stiffness of the nanocomposites. Presented results showed that melt extrusion is an efficient process to obtain anisotropy in thermoplastic GnP-filled nanocomposites, opening this way new application areas of these materials for example as gas-barrier layers or heat sinks with directional control of heat transfer for thermoelectric applications.

## Figures and Tables

**Table 1:** Basic characteristics of the LDPE and graphene nanoplatelets.

xGnP M5 *		
SA (m^2^/g)	120–160	
d_ave_ (µm)	5	
thickness (nm)	6–8	
ρ (g cm^−3^)	2.2	
	**Through plane**	**In-plane**
Thermal conductivity (W/mK)	6	3000
Electrical conductivity (S/m)	102	107
**LDPE**		
*M*_w_	91,641	
*M*_w_/Mn	7.552	
*T*_m_ (°C)	110.62	
*T*_c_ (°C)	94.09	

* Parameters for graphene nanoplatelets from producer data sheet.

## Supplementary Materials

The following are available online at www.mdpi.com/2073-4360/9/7/294/s1.
