# Supplementary material for: Gas Barrier, Thermal, Mechanical and Rheological Properties of Highly Aligned Graphene-LDPE Nanocomposites
Source: Polymers (Basel). 2017 Jul 21;9(7):294. doi: 10.3390/polym9070294 (PMC6432036; doi:10.3390/polym9070294)
Supplement: Supplementary file 1 [file polymers-09-00294-s001.pdf]

polymers

MDPI

Supplementary materials

Thermal properties

The presence of GnP in polymer matrix is expected to influence the crystallization degree of nanocomposites [1,2]. Therefore, the impact of GnP nanofiller on composite crystallinity was examined using Mettler Toledo DSC instrument. DSC analyzes were performed on 2 ÷ 5 mg samples from -50 °C to 150 °C with a scan rate of 10 °C/min in a heating–cooling–heating cycle under N_2 atmosphere. The degree of crystallization was calculated as follows:

$$\chi_c(\%_{crystallinity}) = \frac{\Delta H_m}{\Delta H_0} * 100\%, \quad (S1)$$

Where: ΔH_m - is the melting enthalpy of LDPE (for the composites multiplied by the content of pure LDPE), and ΔH_0 – is a theoretical value of the melting enthalpy of 100% crystalline LDPE. The value ΔH_0 = 293 J/g was used in the crystallinity calculations [3].

Table 1 shows crystallization and melting temperatures determined from DSC thermograms as well as crystallization and melting enthalpies. One can notice that the crystallinity degree reduces slightly, only for sample containing 7.5 wt.% of GnP, whereas for other composites its change is negligible. The same behavior is visible when considering the filler influence on crystallization and melting temperatures.

Table S1. The crystallization and melting parameters determined from DSC thermograms of LDPE-GnP composites.

**Sample**	**Tc(^{0}C) Crystallization temperature**	**ΔHc(J/g) Crystallization enthalpy**	**Tm(^{0}C) Melting temperature**	**ΔHm(J/g) Melting enthalpy**	**χc(%) Crystallinity degree**
**LDPE**	98.2	132.5	110.6	134.5	45.9
**GnP 1wt%**	103.6	124.3	107.9	131.8	45.0
**GnP 3wt%**	103.8	120.6	109.2	133.4	45.5
**GnP 5wt%**	103.8	126.5	108.0	134.6	45.9
**GnP 7.5wt%**	104.0	120.4	108.8	126.9	43.3

The filler content and the degradation temperature of all specimens were studied by means of TGA/DSC 3+ (Mettler Toledo, Inc., Greifensee, Switzerland). TGA measurements were carried out in N_2 atmosphere with a heating rate of 20 °C/min. 2 ÷ 5 mg samples were heated starting from 30 °C up to 900 °C and kept at 900 °C for 10 min in O_2 atmosphere.

TGA analyses have confirmed the content of nanofiller as well as allowed to evaluate the degradation temperature T_d of the nanocomposites. The extracted T_d values, defined as the temperature of 5 % weight loss, are presented in the insert of Figure 1. This parameter is a very important feature of polymeric materials concerning their applications. It is expected that incorporation of fillers with high degradation temperature influences the degradation temperature of nanocomposites. This effect is not visible for 1% and 3% of filler content, however it starts to be visible for samples filled with 5 wt. % and 7.5 wt. % of GnP where the curves are shifted to the higher temperatures.

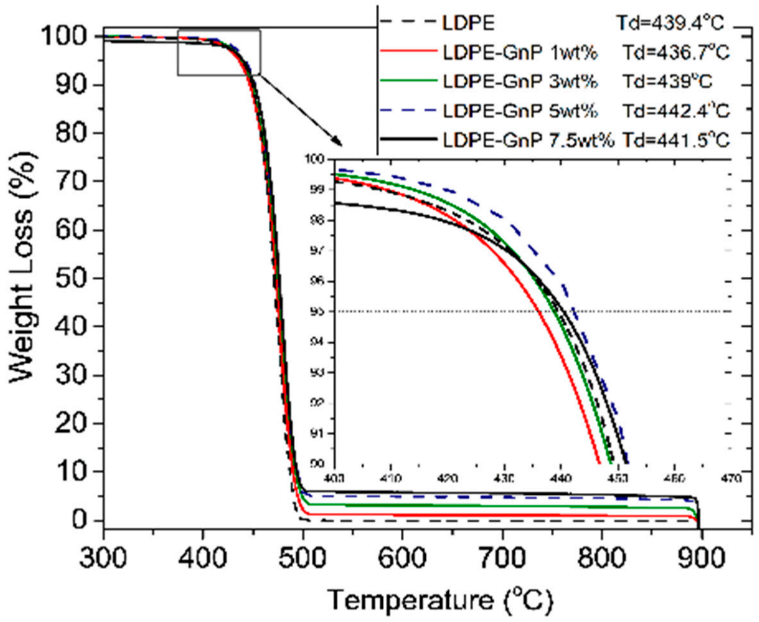

Figure S1. TGA curves measured for investigated nanocomposites. The insert provides deduced degradation temperatures T_d.

References

1. Kalaitzidou, K.; Fukushima, H.; Askeland, P.; Drzal, L.T. The nucleating effect of exfoliated graphite nanoplatelets and their influence on the crystal structure and electrical conductivity of polypropylene nanocomposites. *Journal of Materials Science* **2008**, *43*, 2895-2907.
2. Jiang, X.; Drzal, L.T. Multifunctional high-density polyethylene nanocomposites produced by incorporation of exfoliated graphene nanoplatelets 2: Crystallization, thermal and electrical properties. *Polymer Composites* **2012**, *33*, 636-642.
3. Morawiec, J.; Pawlak, A.; Slouf, M.; Galeski, A.; Piorkowska, E.; Krasnikowa, N. Preparation and properties of compatibilized ldpe/organo-modified montmorillonite nanocomposites. *European Polymer Journal* **2005**, *41*, 1115-1122.
